# Facial Expression Emotion Recognition Model Integrating Philosophy and Machine Learning Theory

**DOI:** 10.3389/fpsyg.2021.759485

**Published:** 2021-09-27

**Authors:** Zhenjie Song

**Affiliations:** School of Humanities and Social Sciences, Xi’an Jiaotong University, Xi’an, China

**Keywords:** facial expression, emotion recognition, philosophy, machine learning, neural networks

## Abstract

Facial expression emotion recognition is an intuitive reflection of a person’s mental state, which contains rich emotional information, and is one of the most important forms of interpersonal communication. It can be used in various fields, including psychology. As a celebrity in ancient China, Zeng Guofan’s wisdom involves facial emotion recognition techniques. His book Bing Jian summarizes eight methods on how to identify people, especially how to choose the right one, which means “look at the eyes and nose for evil and righteousness, the lips for truth and falsehood; the temperament for success and fame, the spirit for wealth and fortune; the fingers and claws for ideas, the hamstrings for setback; if you want to know his consecution, you can focus on what he has said.” It is said that a person’s personality, mind, goodness, and badness can be showed by his face. However, due to the complexity and variability of human facial expression emotion features, traditional facial expression emotion recognition technology has the disadvantages of insufficient feature extraction and susceptibility to external environmental influences. Therefore, this article proposes a novel feature fusion dual-channel expression recognition algorithm based on machine learning theory and philosophical thinking. Specifically, the feature extracted using convolutional neural network (CNN) ignores the problem of subtle changes in facial expressions. The first path of the proposed algorithm takes the Gabor feature of the ROI area as input. In order to make full use of the detailed features of the active facial expression emotion area, first segment the active facial expression emotion area from the original face image, and use the Gabor transform to extract the emotion features of the area. Focus on the detailed description of the local area. The second path proposes an efficient channel attention network based on depth separable convolution to improve linear bottleneck structure, reduce network complexity, and prevent overfitting by designing an efficient attention module that combines the depth of the feature map with spatial information. It focuses more on extracting important features, improves emotion recognition accuracy, and outperforms the competition on the FER2013 dataset.

## Introduction

As an ancient Chinese celebrity, Zeng Guofanno wisdom involves the skill of facial emotion recognition. His book Bing Jian summarizes eight methods on how to identify people, especially how to choose the right one, which means “look at the eyes and nose for evil and righteousness, the lips for truth and falsehood; the temperament for success and fame, the spirit for wealth and fortune; the fingers and claws for ideas, the hamstrings for setback; if you want to know his consecution, you can focus on what he has said.” It is said that a person’s personality, mind, goodness and badness can be showed by his face. That is to say, complete bones are not as neat as skin, clean skin is not as dignified as facial features. Eyes can reflect a person’s good and evil, in contrast to the people who behave indecently, the one with pure and unbiased minds has bright eyes. Therefore, eyes are an important part of facial emotion recognition. Because facial representation (Dubuisson et al., 2002; Sariyanidi et al., 2014; Sun et al., 2018) is an intuitive reflection of human mental state, it contains rich emotional information (D’Aniello et al., 2018; Maydych et al., 2018; Suslow et al., 2020), and can intuitively reflect a person’s true thoughts. In daily human communication, we cannot only convey information through language and text, but we also use some movements and facial expressions to complete the communication between people, and research shows that expressions and movements are often more effective than words. Deliver key messages. Facial expression emotion is a common form of non-verbal communication that can effectively communicate personal emotions and intentions. We can observe other people’s facial expressions with our eyes, and the brain will analyze the data to determine their mental state, completing the expression and communication of emotions between people. Facial expressions can give language emotions, and facial expressions can clearly show a person’s true emotions, which is more accurate than language, in the course of people’s communication.

In social situations, humans will naturally express their personal emotions. An accurate understanding of each other’s emotions will help build mutual understanding and trust. The expression and understanding of emotions is an essential skill for humans. We mainly convey personal emotions in three ways, namely language, voice and facial expressions. Scholars have found that facial expressions are the most important way of expressing human emotion information (Cai and Wei, 2020; Zhang et al., 2021). Facial expression information accounts for about 55 percent of the information transmitted by the experimenters, voice information for 38 percent, and language information accounts for only 7% of the total information. It’s clear that, when compared to language and sound, facial expression information is more important for emotional comprehension. Naturally, researchers concentrate on facial expressions in order to gain a better understanding of human inner emotional activities.

In recent years, as computers have increasingly powerful computing power and huge data sets continue to emerge, machine learning algorithms (Domínguez-Jiménez et al., 2020; Zhang et al., 2020; Cai et al., 2021a) have developed vigorously. Compared with traditional methods, the machine learning algorithm integrates the two processes of feature extraction (Koduru et al., 2020) and classification (Oberländer and Klinger, 2018), reduces the operation process, and can automatically extract the internal features of the sample data, has powerful feature extraction capabilities, and is related to computer vision (CV) (Schmøkel and Bossetta, 2021; Cai et al., 2021b). The performance in various competitions is very good. Among them, Convolutional Neural Network (CNN) (Santamaria-Granados et al., 2018; Ghosal et al., 2019; Gao et al., 2021) is one of the most common machine learning algorithms, and the classification effect of images is excellent, and the recognition accuracy rate of the ImageNet database has not been updated. Therefore, many researchers have begun to use neural networks (Chu et al., 2021; Tong et al., 2021) to solve the recognition problem of facial expressions. However, the facial expression images collected in real life are very uncontrollable, and these uncontrollability increase facial expressions. How to design the structure of the CNN to efficiently and accurately recognize facial expressions still needs continuous exploration.

Based on the above observations, I found that how the CNN can efficiently and accurately recognize facial expressions is the focus of this article. Therefore, I propose a dual-channel emotion recognition algorithm. The first path of the proposed algorithm uses the Gabor feature of the ROI area as input. In order to make full use of the detailed features of the active facial expression area, first segment the active facial expression area from the original face image, use Gabor transform to extract the features of this area, and focus more on the detailed description of the local area. The second path proposes an efficient channel attention network based on deep separable convolution to achieve linear bottleneck structure improvement, reduce network complexity and prevent overfitting, and improve the accuracy of emotion recognition.

The main contributions of this paper are as follows:

(1) This paper proposes a novel feature fusion dual-channel expression recognition algorithm based on machine learning theory and philosophical thinking. It has achieved competitive performance on the FER2013 data set and has a positive significance in promoting the recognition and employment of people.
(2) Aiming at the problem that the features extracted using CNNs ignore the subtle changes in the active areas of facial expressions, the first pass of the proposed algorithm takes the Gabor features of the ROI area as input. In order to make full use of the detailed features of the active areas of facial expressions, start the original face image is segmented into the active area of expression, the Gabor transform is used to extract the features of this area, and the detailed description of the local area is focused.
(3) An efficient attention module is designed to combine the depth of the feature map with the spatial information, focus on the extraction of important features, and use the joint loss function to make the network have a better feature discrimination effect, reduce the difference of the same facial expressions within the class, expand the feature spacing between different facial expressions, and ensure the accuracy of classification.

The rest of this paper is arranged as follows. In section “Related Work,” we introduce relevant work, in section “Methodology,” we describe the algorithm in this paper, and in section “Experiments and Results,” we give experiments and experimental results. Section “Conclusion” presents the research conclusions of this paper.

## Related Work

### Emotion Recognition Based on Facial Expressions

The process of human communication is inextricably linked to the fluctuation of various emotions. When people are experiencing basic emotions, their faces will display a variety of expression patterns, each with its own set of characteristics and distribution scale. Facial expression recognition is a crucial part of human-computer interaction that allows computers to understand facial expressions based on human thinking. According to the processing of facial expression recognition process can be divided into three important face detection, feature extraction and classification module, face detection as the key technology of face recognition (Adjabi et al., 2020; Zhang et al., 2021) with its rapid development has basic mature, which can effectively extracted from the original face image of excellent characteristics and the characteristics of correct classification becomes key factor affecting the recognition result. For example, Gao and Ma (2020) obtained facial expression attributes from facial images so as to predict emotional states according to facial expression changes.

### Speech-Based Emotion Recognition

Language is another way for human beings to express emotions. The speech signals expressed by human beings in different emotional states have different characteristics and rules, such as speed, pitch, duration, etc. The emotion recognition method based on speech is to identify and judge the emotional information of the speaker at this time by studying and analyzing the physical characteristics of the speaker’s speech in different emotional states. Ton-That and Cao (2019) applied speech signals to emotion recognition and achieved good results on the voice emotion database. However, on the one hand, individual differences will lead to great differences in speech signals, which requires the establishment of a large phonetic database, which will bring some difficulties to recognition. On the other hand, the noisy environment will affect the sound quality of speech, thus affecting the emotion recognition, so the acquisition of speech signal has a high requirement on the surrounding environment.

### Emotion Recognition Based on Physiological Signals

The basis of emotion recognition based on physiological signals is that humans will produce different responses under different stimuli. For example, physiological signals such as brain electricity, electrocardiogram, pulse, and skin electrical response can all reflect emotions. Momennezhad (2018) used EEG signals for emotion recognition, extracting features from the time domain and frequency domain of EEG signals. Although the changes of physiological signals are not controlled by humans, they can most objectively reflect human emotional conditions.

### Emotion Recognition Based on Gestures

People will involuntarily undergo some posture changes in different environmental states and moods, and judge human emotions based on physical information such as the time and frequency of these posture changes, according to gesture-based emotion recognition. Ajili et al. (2019) used human movement analysis to identify motion, then evaluated and evaluated the emotions expressed by human motion posture. However, the single use of human gestures for emotion recognition has certain limitations, because many gestures do not have emotional significance or the same gestures have different emotional meanings in different background environments, so human gestures are usually different from others. The modalities (such as expressions, speech, etc.) are combined for emotion recognition.

Expressions are the most intuitive way to convey emotions among several ways to express human emotion information, such as facial expressions, voices, physiological signals, and gestures, and expression information is relatively easy to obtain in most environments, so I use them. Human emotional states are studied using facial expressions as objects.

## Methodology

### The Philosophical Definition of Emotion

Psychology defines emotion as: “a special form of reflection of human beings on objective reality is the experience of human attitudes toward whether objective things meet human needs.” It can be understood from this definition that emotion is a subjective experience, subjective attitude, or subjective reflection, which belongs to the category of subjective consciousness, not the category of objective existence. Dialectical materialism believes that any subjective consciousness is a reflection of a person’s objective existence. Emotion is a special subjective consciousness and must correspond to a special objective existence. The key to the problem lies in whether such a special objective existence can be found. It is not difficult to find that “whether a person’s objective things meet people’s needs” is actually a typical value judgment problem, “meeting people’s needs” is the value characteristic of things, an objective existence, “attitude” and “experience” Both are the way people recognize or reflect the value characteristics of things. In this way, the psychological definition of emotion can be expressed as: “Emotion is the subjective reflection of people on the value characteristics of things.” The objective existence corresponding to emotion should be the value characteristic of things. From this I can get the philosophical definition of emotion: emotion is the subjective reflection of human beings on the value relationship of objective things.

### Two-Channel Emotion Recognition Model

This section will elaborate on the proposed dual-channel emotion recognition model from the ROI area division and Gabor feature of the first path, and the efficient channel attention network of the second path.

#### Division of ROI Area

Let _*P_el_*_ and _*P_er_*_ denote the positions of the left and right eyes, _*P_n_*_ denote the key points of the tip of the nose, _*P_ml_*_ and _*P_mr_*_ denote the key points of the left and right corners of the mouth, _*X*_1__ and _*X*_2__ denote the horizontal coordinates of the left and right borders of the face, and _*Y*_1__ and _*Y*_2__ denote the vertical positions of the upper and lower edges, respectively, coordinate. Assuming that the position of the eyebrow area is calculated as an example, only the height and width of the area need to be calculated. The calculation equation for the height of the eyebrow area is as follows:


(1) $${\text{H}}_{\text{eye}}=\left\{ \begin{array}{c} \frac{\left|{\text{P}}_{\text{el}}:y-{\text{P}}_{\text{n}}:y\right|}{2}+|{\text{Y}}_{2}-{\text{P}}_{\text{er}}:y|,p_{\text{el}}:y\leq {\text{P}}_{\text{er}}:y\\ |{\text{Y}}_{2}-{\text{P}}_{\text{el}}:y|+\frac{\left|{\text{P}}_{\text{er}}:y-{\text{P}}_{\text{n}}:y\right|}{2},P_{\text{el}}:y\geq {\text{P}}_{\text{er}}:y \end{array}\right.$$

I use _*W_eye_*_ to represent the width of the eyebrow region. In order to make the extracted human eye region not only include the eye, but also the part of the information around the corner of the eye, the calculation of _*W_eye_*_ directly takes the distance between the left and right edges. The calculation equation is as follows:


(2) $$W_{\text{eye}}=\left|X_{2}-X_{1}\right|$$

Similarly, the calculation of the position of the mouth in the face image is as follows, using _*H_mouth_*_ and _*W_mouth_*_ to represent the height and width of the region, respectively.


(3) $${\text{H}}_{\text{mouth}}=\left\{ \begin{array}{c} \frac{\left|{\text{P}}_{\text{n}}:y-{\text{P}}_{\text{ml}}:y\right|}{2}+|{\text{P}}_{\text{mr}}:y-Y_{1}|,p_{\text{ml}}:y\leq {\text{P}}_{\text{mr}}:y\\ |{\text{P}}_{\text{ml}}:y-Y_{1}|+\frac{\left|{\text{P}}_{\text{n}}:y-{\text{P}}_{\text{mr}}:y\right|}{2},P_{\text{ml}}:y\geq {\text{P}}_{\text{mr}}:y \end{array}\right.$$

(4) $$W_{\text{mouth}}=\left|X_{2}-X_{1}\right|$$

Finally, the rectangular clipping areas of the eyebrow, eye, and mouth in the face image are determined using the above calculation method. The interference of non-key parts of the face can theoretically be reduced, and the calculation cost can be reduced, by cutting out these regions with large facial expression changes.

#### Gabor Filter

Compared with other wavelets, Gabor transform has a unique biological background. The Gabor filter is similar to the frequency and direction representation of the human visual system in terms of frequency and direction, and can extract local information of different frequencies, spatial positions and directions of the image. A special advantage of Gabor filters is their invariance to scale, rotation and translation. The reason why Gaboe wavelet can be used for facial expression recognition is that when expression changes occur, the key parts of the face such as eyes, mouth, and eyebrows will undergo great changes due to muscle changes. These parts are reflected in the image as gray-scale changes. Severe, the real and imaginary parts of the wavelet will fluctuate at this time, so the amplitude response of the Gabor filter in these parts will be very obvious, so it is very suitable for extracting local features of expressions. In the field of image processing, two-dimensional Gabor filtering is generally used to process images. The kernel function of the two-dimensional Gabor wavelet can be written as:


(5) $$\psi_{\text{uv}}\left(\text{z}\right)=\frac{{||{\text{k}}_{\text{uv}}||}^{2}}{\sigma^{2}}\times {\text{e}}^{\frac{{||{\text{k}}_{\text{uv}}||}^{2}{||\text{z}||}^{2}}{2\sigma^{2}}}\times \left({\text{e}}^{{\text{ik}}_{\text{uv}}\text{z}}-e^{\frac{\sigma^{2}}{2}}\right)$$

where _*u*_ and _*v*_ represent the direction and frequency of the Gabor wavelet kernel, _*z*=(*x,y*)_ represents the position of a certain pixel in the image, _σ_ represents the filter bandwidth, and _|k_uv_|^2^/σ^2^_ is used to compensate for the attenuation of the energy spectrum determined by the frequency.

The Gabor feature of the facial expression image can be obtained by convolving the facial expression image and the Gabor wavelet kernel. Assuming that the gray value of the (x, y) point in the facial expression image is set to k, the calculation equation for the Gabor feature is as follows :


(6) $$G_{uv}\left(x,y\right)=I\left(x,y\right)\;*\;\psi_{uv}\left(x,y\right)$$

where _*G_uv_*(*x,y*)_ represents the Gabor feature of the extracted image, _ψ_*uv*_(*x,y*)_ represents the kernel function of the two-dimensional Gabor wavelet, and * represents the convolution operation.

#### Feature Fusion

**FIGURE 1:**
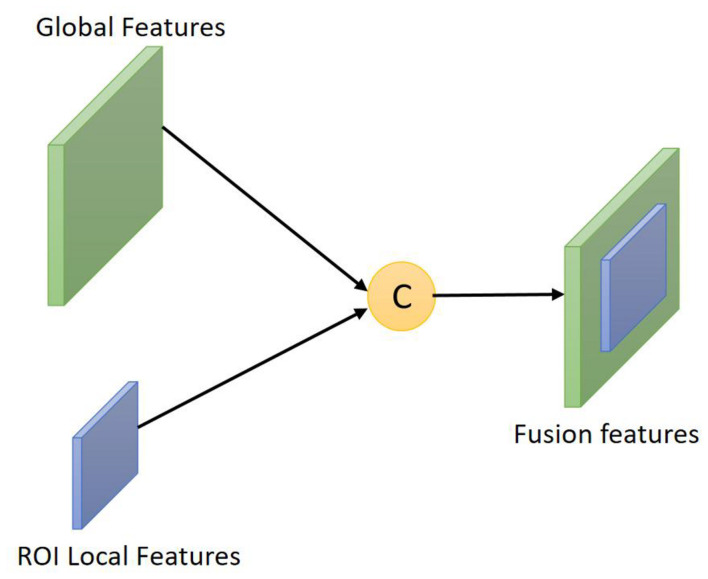
Schematic diagram of feature fusion.

To make full use of the features of the key areas of facial expressions and make up for the lack of global representation in the local features extracted by Gabor, the features extracted by CNN and the local features of the ROI region extracted by Gabor are feature-fused. Simply put, feature fusion is to combine multiple different features extracted by different algorithms into a new feature with stronger characterization capabilities through a certain fusion method. The process of feature fusion is shown in Figure 1.

There are three ways of feature fusion, namely summation, product and splicing. Among the three feature fusion methods, the method of splicing and fusion is simple in calculation and less computationally expensive. Therefore, this article uses splicing and fusion to compare Gabor features and CNN. Feature fusion is performed, and the local features of the ROI region extracted by Gabor and the features extracted by CNN are fused in the fully connected layer of the CNN. Suppose two feature vectors with the same dimension are defined as _*X*=(*x*_1_,*x*_2_,⋯,*x_n_*)_ and Y = 1, then the calculation equation for feature stitching is as follows:


(7) $$Z=\left(x_{1},x_{2},\cdots ,x_{n},y_{1},y_{2},\cdots ,y_{n}\right)$$

#### Channel Attention Model

To extract more core expression features from the facial expression feature map, I introduces the ultra-lightweight attention module ECA-Net (Wang et al., 2020) to weight the attention of the improved linear bottleneck structure, and give greater weight to the core features, Which makes the network pay more attention to the core features of expressions. This structure contains only one parameter k, but the performance improvement it brings is very obvious. The main function of this module is to generate weights for each channel and learn the correlation between features, just like humans always selectively ignore non-critical information, but instead focus on information that is useful to us. This module The purpose is to let the network model ignore some non-core features, increase the emphasis on core features, and the module only adds a very small amount of additional parameters.

**FIGURE 2:**
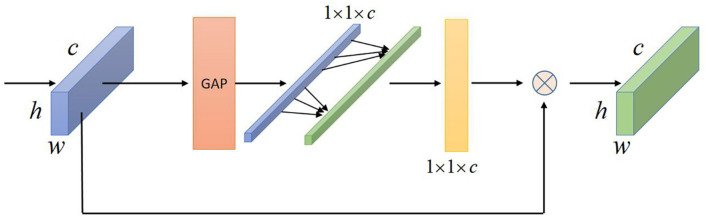
Schematic diagram of the channel attention model.

As shown in Figure 2, the distribution of features in the spatial dimension is compressed and extracted from a two-dimensional matrix to a single value, and this value obtains the feature information in this space. Then through a fully connected layer to complete the channel dimension reduction, and then through the second fully connected layer to complete the channel dimensionality, so as to obtain the relevant dependencies between the various channels, generate weights for each feature channel, and comprehensively obtain one of the feature channels. For the correlation between the channels, the important channel features of facial expressions are generated with larger weights, and on the contrary, smaller weights are generated, that is, the attention mechanism is introduced. It generates channel weights through local one-dimensional convolution in high dimensions, and obtains the correlation dependency between each channel. The side effect of channel dimension reduction on the direct correspondence between channels and weights is avoided, and obtaining appropriate cross-channel correlation dependencies is more efficient and accurate for establishing the channel attention mechanism. The last two attention modules both multiply the weights of the generated channels to the original input feature map, and merge the features weighted by attention with the original features to complete the feature attention weighting in the channel space.

#### Overall Structure

**FIGURE 3:**
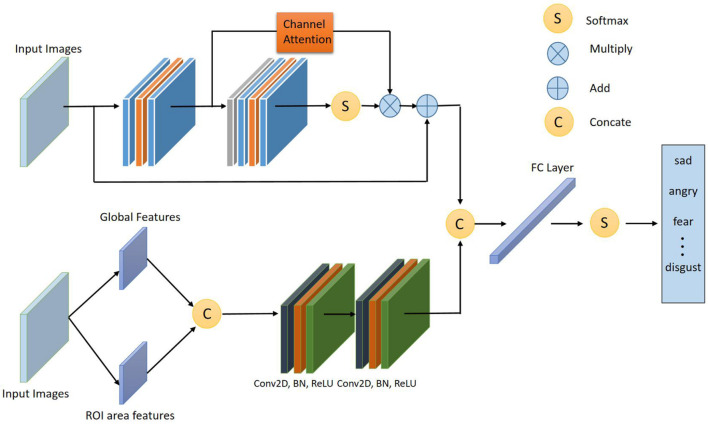
Schematic diagram of the overall framework of the proposed algorithm.

Figure 3 shows the overall frame diagram of this algorithm. Firstly, the feature extraction module is composed of two different CNN branches: The first CNN branch takes the Gabor feature as the input. In order to make full use of the features of the regions with obvious facial expression changes and rich facial information, the original face image should be preprocessed and the emotion-related ROI region should be trimmed out, and then Gabor wavelet changes should be used to extract the ROI feature. Since the extracted features still have a high dimension, feature mapping processing of Gabor features is required before feature fusion. This CNN is composed of two convolution layers, which reduces the size of Gabor features and facilitates subsequent feature fusion. In the second path, an efficient channel attention network based on deep separable convolution is proposed to improve the linear bottleneck structure, reduce network complexity and prevent overfitting. By designing an efficient attention module, the depth of the feature map is combined with spatial information, focusing more on the extraction of important features, and improving the accuracy of emotion recognition. Finally, the feature classification module classifies the fused features through Softmax layer.

## Experiments and Results

### Experimental Setup

The research in this paper is carried out on the PC platform, and the experiment is carried out on the ubuntu18.04 operating system. The experiment is based on the Pytorch deep learning framework, and the programming language uses Python 3.6.5. The hardware platform is: Intel Core i7-9700k CPU, 16GB memory, GPU is GTX1080TI, video memory is 11GB. In order to ensure the fairness of the experiment on the improved network and the comparison network, the training parameters used in the experiment are exactly the same. All model training strategies adopt the learning rate attenuation strategy. The initial learning rate is 0.01 and the attenuation coefficient is 0.0001, the batch size is 128. After the model training is completed, all images in the training data set are called an epoch, and 150 epochs are set in the experiment. In order to optimize the network faster, I use the Adam optimization algorithm.

### Experimental Data Set

The data set used in this article is FER-2013. Due to the small amount of data in the original facial expression data set, it is far from enough for data-driven deep learning, so data augmentation is a very important operation. In the network training stage, in order to prevent the network from overfitting, I first do a series of random transformations, including flipping, rotating, cutting, etc., and then transform the data image size to 104 × 104 size, and then randomly cut into 96 × 96 size Image, and then randomly rotate the image between 0 and 15° and perform horizontal mirroring operation, and then send it to the network for training. In the network test stage, we cut the four corners and the center of the image to obtain five 96 × 96 images, and then perform the horizontal mirroring operation, respectively, which is equivalent to amplifying the data by 10 times. I input the amplified picture into the network for its recognition, and average the results, and finally the output classification with the highest score is the corresponding expression. This method can expand the size of the data set, make the trained network model more generalized and robust, and further improve the accuracy of recognition.

### Evaluation Method

The overall accuracy rate is used as the evaluation index of this study, and its calculation formula is as follows:


(8) $$Acc=\frac{TP+TN}{TP+TN+FP+FN}$$

where TP represents the positive samples predicted by the model as positive, TN represents the negative samples predicted by the model as negative, FP represents the negative samples predicted by the model as positive, and FN represents the positive samples predicted by the model as negative.

### Experimental Results

In order to verify the reliability of the overall algorithm in this paper, this paper has carried out comparative experiments on the FER-2013 data set with the current advanced expression recognition network to evaluate the performance of the algorithm in this paper. The experimental results are shown in Table 1.

**TABLE 1:** Comparison of experimental results with different methods.

Methods	Acc
InceptionV4 (Szegedy et al., 2017)	0.7080
DNNRL (Kim et al., 2016)	0.7082
ICL (Liu and Zhou, 2020)	0.7215
ABP (Liu et al., 2019)	0.7316
MobileNetV3	0.7189
**Ours**	**0.7400**

*The bold values represent the best values of the adopted methods.*

Table 1 is a comparison of the recognition rates of different methods. This article uses a lightweight network structure. When compared with MobileNetV3 and Inception, which are also lightweight networks, the accuracy of the FER-2013 data set is improved with fewer model parameters. Increased by 3.3 and 4.7%. Compared with the current mainstream methods on the FER-2013 data set, DNNRL (Kim et al., 2016) proposed combining multiple CNNs and using weighted joint decision-making methods, and ICL (Liu and Zhou, 2020) proposed clustering to obtain the center distance of expression classes and continuously Adding the method of difficult sample training and the method of combining bilinear pooling and attention mechanism proposed by ABP (Liu et al., 2019), the method in this paper has achieved superior performance and achieved a higher recognition rate of 74.00%.

In order to avoid misjudgment of model performance when only the overall recognition rate is used as the evaluation index, we conduct detailed experiments on the recognition results of each type of expression through the confusion matrix. The confusion matrix is also called the error matrix. Each row represents the expression prediction label, and each column represents the actual expression label. Using the confusion matrix can clearly observe the recognition of each type of data, and from the recognition accuracy of each type of expression, we can analyze the performance of the network model in more detail.

Table 2 is the confusion matrix of the high-efficiency channel attention model proposed in this paper for the recognition results of the FER-2013 test set. The data in bold on the diagonal line in the table represents the recognition accuracy of each type of expression correctly classified, and the remaining data are expression errors. The proportion of classification, the last line is the average recognition accuracy of all expressions that are correctly classified. For example, the neutral expression recognition accuracy in the lower right corner of the diagonal of the confusion matrix is 0.77, which indicates that 76% of the expression samples in the expression data set are correctly predicted. It can be seen that the recognition accuracy of happy and surprised expressions is high, with accuracy rates of 0.91 and 0.83, respectively, while the recognition accuracy of disgusted and angry expressions is low, with accuracy rates of only 0.64 and 0.66. By observing the samples, it is found that there are indeed many similarities between the facial morphology of fear and surprise, anger and disgust, and the number of samples is far smaller than the number of happy and surprised, which leads to the model’s insufficient learning of its features, so the recognition rate is low. Finally, the average recognition rate of the model in the FER-2013 test set reached 0.74.

**TABLE 2:** Confusion matrix of recognition rate on FER-2013 dataset.

	Anger	Fear	Disgust	Happy	Sad	Surprised	Normal
Anger	**0.66**	0.01	0.11	0.04	0.11	0.01	0.06
Fear	0.20	**0.69**	0.04	0.05	0.00	0.00	0.02
Disgust	0.09	0.00	**0.64**	0.02	0.12	0.05	0.09
Happy	0.01	0.00	0.02	**0.91**	0.02	0.01	0.03
Sad	0.05	0.00	0.11	0.05	**0.68**	0.00	0.13
Surprised	0.02	0.00	0.06	0.05	0.02	**0.83**	0.02
Normal	0.03	0.00	0.03	0.05	0.12	0.01	**0.77**
Acc	**0.74**						

*The bold values represent the best values of the adopted methods.*

### Ablation Experiment for Feature Fusion

To verify the influence of the feature fusion strategy on the performance of the proposed algorithm, an ablation experiment is set up in this section, where add represents the feature addition strategy, mul represents the feature multiplication strategy, and C represents the feature concat strategy. The results of the ablation experiment are shown in Table 3.

**TABLE 3:** Results of ablation experiments with feature fusion.

Methods	Acc
Add	0.7285
Mul	0.7195
**C**	**0.7400**

*The bold values represent the best values of the adopted methods.*

It can be seen from Table 3 that the feature concat strategy has achieved the best results. In addition, the feature addition strategy is better than the feature multiplication strategy. Therefore, this proves that the proposed algorithm is effective in adopting the feature concat strategy.

### Ablation Experiment for Attention Model

To verify the influence of the channel attention mechanism on the performance of the proposed algorithm, an ablation experiment is set up in this section. CA stands for the channel attention mechanism and SA stands for the spatial attention mechanism. The results of the ablation experiment are shown in Table 4.

**TABLE 4:** Results of ablation experiments with attention.

Methods	Acc
SA	0.7395
**CA**	**0.7400**

*The bold values represent the best values of the adopted methods.*

It can be seen from Table 4 that the channel attention mechanism has achieved better performance, which proves the superiority of CA in facial emotion recognition.

## Conclusion

In this paper, I propose a novel feature fusion dual-channel expression recognition algorithm based on machine learning theory and emotional philosophy. Because features extracted using CNNs ignore subtle changes in the active regions of facial expressions, the proposed algorithm’s first path takes the Gabor feature of the ROI region as input. The active facial expression region is first segmented from the original face image, and the features of this region are extracted using Gabor transform, focusing more on the detail description of the local region, in order to make full use of the detail feature of the active facial expression region. To improve the linear bottleneck structure, reduce network complexity, and avoid overfitting, a channel attention network based on deep separable convolution is proposed in the second path. The depth of the feature map is combined with spatial information by designing an efficient attention module, focusing more on the extraction of important features and improving the accuracy of emotion recognition. On the FER2013 data sets, competitive performance was achieved. Furthermore, this research will serve as a guide for promoting people selection, and it also confirms that Zeng Guofan’s philosophy of employing people is effective.

In future work, we will investigate the feasibility of real-time face recognition, and will use the Internet of Things technology to collect faces in real time for emotion recognition.

## Data Availability Statement

Publicly available datasets were analyzed in this study. This data can be found here: https://www.kaggle.com/c/challenges-in-representation-learning-facial-expression-recognition-challenge/data/.

## Author Contributions

ZS was responsible for designing the framework of the entire manuscript, from topic selection to solution to experimental verification.

## Conflict of Interest

The author declares that the research was conducted in the absence of any commercial or financial relationships that could be construed as a potential conflict of interest.

## Publisher’s Note

All claims expressed in this article are solely those of the authors and do not necessarily represent those of their affiliated organizations, or those of the publisher, the editors and the reviewers. Any product that may be evaluated in this article, or claim that may be made by its manufacturer, is not guaranteed or endorsed by the publisher.
